# SARS-CoV-2-associated encephalitis: arguments for a post-infectious mechanism

**DOI:** 10.1186/s13054-020-03389-1

**Published:** 2020-11-23

**Authors:** Adrien Picod, Vera Dinkelacker, Julien Savatovsky, Pierre Trouiller, Antoine Guéguen, Nicolas Engrand

**Affiliations:** 1 Neuro-Intensive Care Unit, Fondation Ophtalmologique Adolphe de Rothschild, 29 rue Manin, 75019 Paris, France; 2 Department of Neurology, Fondation Ophtalmologique Adolphe de Rothschild, 29 rue Manin, 75019 Paris, France; 3 Department of Radiology, Fondation Ophtalmologique Adolphe de Rothschild, 29 rue Manin, 75019 Paris, France

Dear editor,

Patients with severe acute respiratory syndrome coronavirus 2 (SARS-CoV-2) infection may experience neurological symptoms [1–3] which remain poorly understood and could correspond to multiple pathophysiological mechanisms. We herein report a case in which neurological symptoms appeared delayed from the respiratory infection and suggested a post-infectious encephalitis.

In April 2020, a 58-year-old woman with a history of hypertension and chronic kidney disease was admitted to our intensive care unit after two neurological episodes. The first was a clonic seizure followed by persistent aphasia and right-sided hemiparesis, for which she was admitted to a general hospital. The next day, she developed an unexplained coma requiring transferal to our hospital. Upon arrival, she was comatose, unresponsive to verbal and nociceptive stimulation with diffuse hyperreflexia and showed tremulation of both arms. Pupillary assessment was normal, and body temperature was 37.3 °C. Concerns about airway protection prompted the intubation decision. Although brain CT scanner was normal, thoracic CT scan revealed bilateral patchy ground glass opacities suggestive of a mild coronavirus disease 2019 (COVID-19). Brain MRI showed bilateral lesions suggestive of encephalitis (Fig. 1). Serial EEG was performed and showed diffuse intermittent periodic activity predominating in derivations matching with the localization of MRI lesions (Fig. 2a). At that time, the absence of epileptic activity despite ongoing tremulations of both arms suggested that tremulations represented myoclonus of subcortical origin. Lumbar puncture revealed no intracranial hypertension, and CSF analysis showed no meningitis (WBC 2/mm^3^, proteins 0.28 g/L without oligoclonal bands) nor infectious agent (including negative PCR for *herpesviridae*), allowing the discontinuation of empiric therapy with acyclovir, cefotaxime and amoxicilline. Search for antibodies targeting intracellular and neuronal cell surface antigens was negative on serum and CSF. Elevated interleukin-6 at 723 pg/mL was found in CSF, contrasting with a moderately elevated serum level of 106 pg/mL (reference range 0–7 pg/mL). On day (D) 4, clinical deterioration with myoclonus worsening prompted the insertion of a left retrograde jugular catheter for brain oxygenation monitoring which showed profound desaturation (SjvO_2_ 47%) that persisted despite deep sedation and attempts to increase cerebral oxygen delivery with initiation of vasopressors and red blood cells transfusion, consistent with an increased cerebral metabolic rate. Considering the negative search for infectious agents and the hypothesis of an inflammatory encephalitis, corticotherapy was initiated by daily 250 mg pulses of methylprednisolone for 3 days, followed by 1 mg/kg/day. Evolution was gradually favorable with jugular resaturation over 70% within a day and progressive normalization of the EEG (Fig. 2b). The patient was successfully extubated on D12 with mild short-term memory impairment and otherwise normal neurological examination. She was discharged from the ICU on D17. During hospitalization, search for SARS-CoV-2 RNA was repeatedly negative in respiratory (D1, D3, D10), feces (D3) or CSF (D1, D3) samples. However, both anti-SARS-CoV-2 IgM and IgG were positive in serum but not in CSF (ELISA, paired samples taken on D10).

**Fig. 1:**
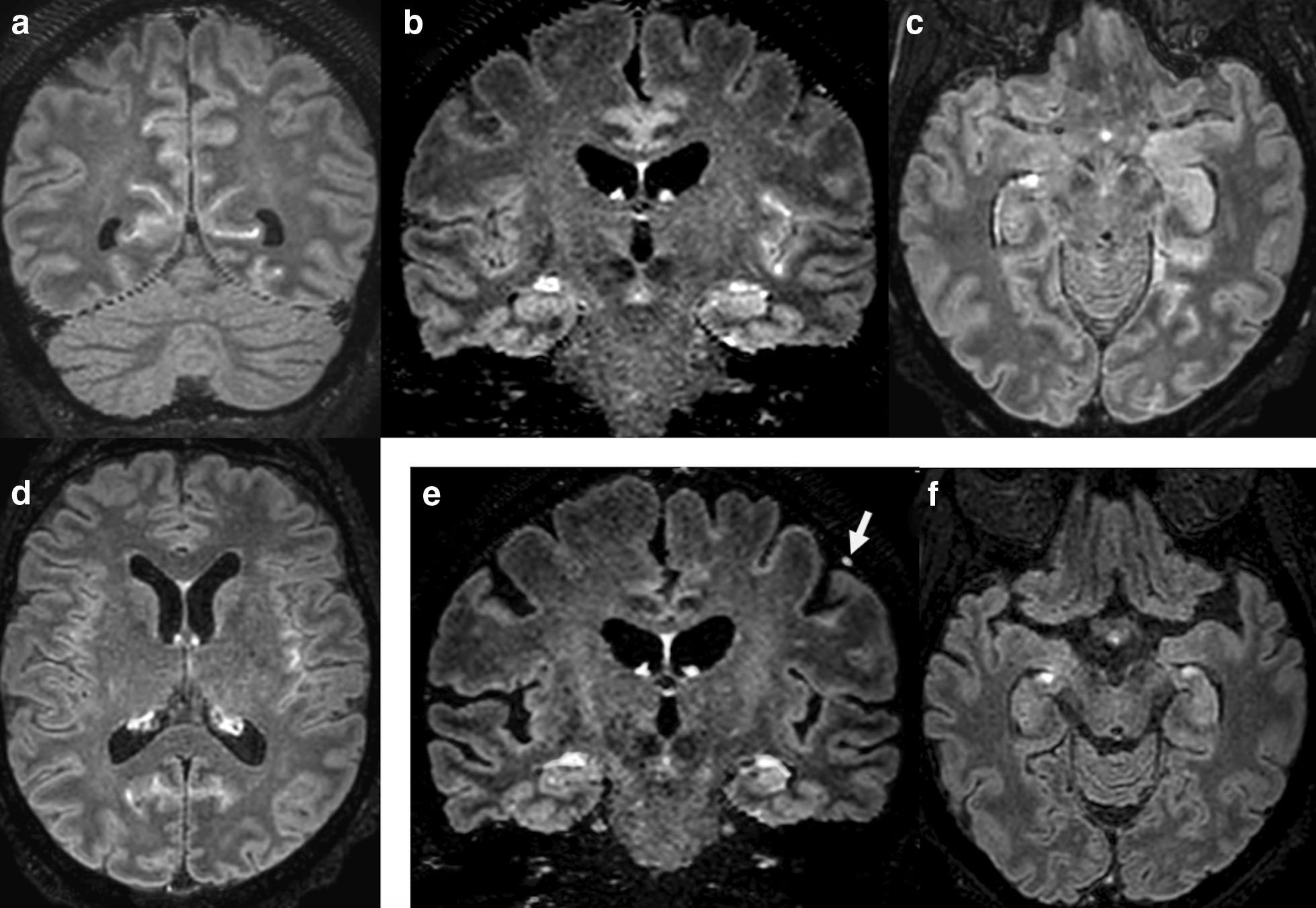
Brain MRI. Initial coronal (**a**, **b**) and axial (**c**, **d**) fluid-attenuated inversion recovery postcontrast MRI displaying hypersignal or enhancement of meninges, cortical and subcortical regions spread over the insula, the cingula, the medial part of the occipital areas and the internal part of the left temporal lobe. 10-week follow-up MRI (**e**, **f**) demonstrates complete disappearance of leptomeningeal and cortical FLAIR hyperintensities and enhancements, except for a subtle left-sided residual nodular leptomeningeal enhancement (arrow) adjacent to a cortical vein

**Fig. 2:**
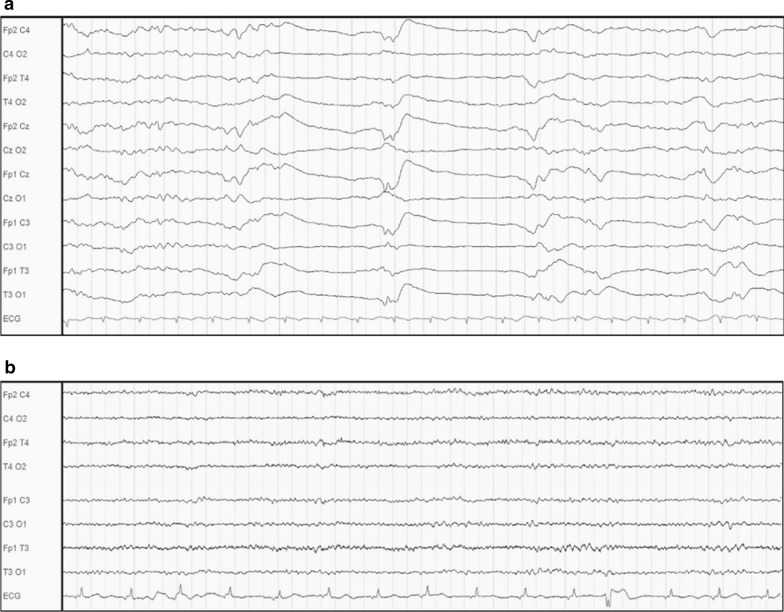
Electroencephalographic findings. On admission, the patient’s EEG showed intermittent slow periodic activity predominant on the vertex and left hemisphere (**a**, 04/20/20), reactive to stimulation. Two weeks later (**b**, 05/04/20), both EEG and neurological status had nearly fully recovered. Recording features: longitudinal montage (with vertex electrode in **a**), 10 s per page, 10 µV/mm, 70 Hz high and 0.3 Hz low-frequency filters

Several mechanisms could explain neurological manifestations associated with SARS-CoV-2, including direct neuroinvasion, para- or post-infectious immunological disorder, potential neurotoxicity of therapies and toxic/metabolic encephalopathy, especially in the context of high burden of proinflammatory cytokines that characterizes severe COVID-19 [4–6]. At the time of hospitalization, our patient was exempt from respiratory symptoms. Although direct search of viral RNA was negative, a positive IgM serology suggested a recent SARS-CoV-2 infection. Furthermore, focal lesions and substantially higher CSF interleukine-6 level as compared with serum sample argue for an autochthonous brain inflammatory process rather than a toxic encephalopathy induced by passive transfer of proinflammatory cytokines from the systemic compartment [3]. Finally, the efficacy of immunomodulation with corticosteroids supports an immunological mechanism.

Taken together, these findings suggest that SARS-CoV-2 could trigger a post-infectious inflammatory encephalitis.

## Data Availability

All data analyzed in this report are available on simple request to the corresponding author.

## Abbreviations

SARS-CoV-2: Severe acute respiratory syndrome coronavirus 2
COVID-19: Coronavirus disease 2019
D: Day
